# Targeting of Proteins for Translocation at the Endoplasmic Reticulum

**DOI:** 10.3390/ijms23073773

**Published:** 2022-03-29

**Authors:** Martin R. Pool

**Affiliations:** School of Biological Science, Faculty of Biology, Medicine and Health, University of Manchester, Manchester M13 9PL, UK; martin.r.pool@manchester.ac.uk; Tel.: +44-161-275-5392

**Keywords:** endoplasmic reticulum, ribosome, signal sequence, signal recognition particle, protein targeting, GET, SND, Sec61 translocase, NAC

## Abstract

The endoplasmic reticulum represents the gateway to the secretory pathway. Here, proteins destined for secretion, as well as soluble and membrane proteins that reside in the endomembrane system and plasma membrane, are triaged from proteins that will remain in the cytosol or be targeted to other cellular organelles. This process requires the faithful recognition of specific targeting signals and subsequent delivery mechanisms to then target them to the translocases present at the ER membrane, which can either translocate them into the ER lumen or insert them into the lipid bilayer. This review focuses on the current understanding of the first step in this process representing the targeting phase. Targeting is typically mediated by cleavable N-terminal hydrophobic signal sequences or internal membrane anchor sequences; these can either be captured co-translationally at the ribosome or recognised post-translationally and then delivered to the ER translocases. Location and features of the targeting sequence dictate which of several overlapping targeting pathway substrates will be used. Mutations in the targeting machinery or targeting signals can be linked to diseases.

## 1. Targeting Signals

**Figure 1:**
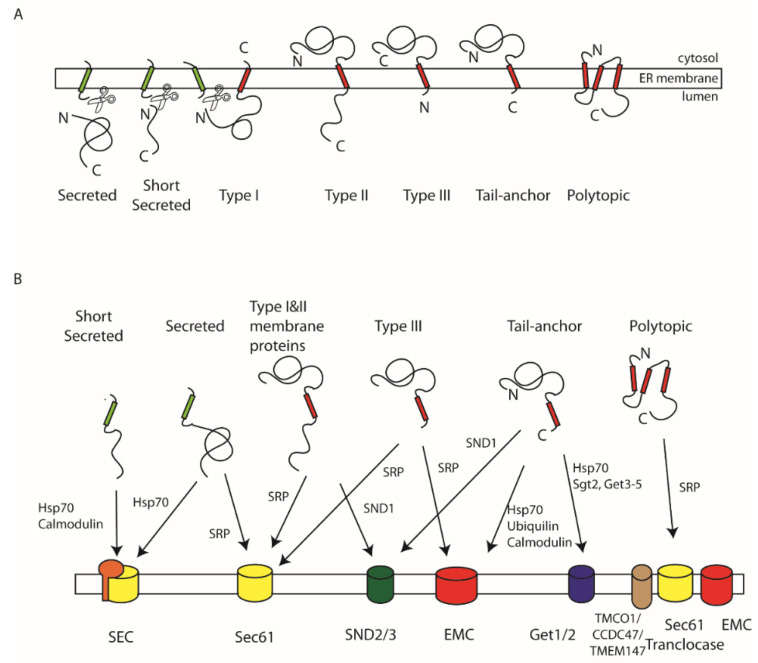
Targeting signals, pathways, and translocases: (**A**) Soluble secretory and luminal proteins as well as type I membrane proteins are targeted by cleavable N-terminally located signal sequences (green). Type II, type III and tail-anchor membrane proteins as well as multi-spanning polytopic membrane proteins utilise internal TM domains (red) for targeting. (**B**) These different classes of secretory and membrane proteins are translocated across or inserted into the membrane by a number of translocases with overlapping specificity. Short secretory proteins are translocated by the SEC translocase and maintained in a translocation-competent state by Hsp70 chaperones or calmodulin. Longer secretory proteins can use the same pathway as well as delivery to the Sec61 translocase by SRP. Type I and II membrane proteins can also be delivered to Sec61 by SRP. Those with more central and C-terminal TM domains can also be targeted by the SND pathway. Type III membrane proteins can be delivered by SRP to either Sec61 or the EMC translocase. Tail-anchored membrane proteins can utilise the SND pathway and the GET pathway as well as delivery to the EMC complex involving Hsp70, calmodulin, or ubiquilins. Finally, polytopic membrane proteins are targeted to the Sec61 translocon by SRP and may recruit the EMC or TMCO1 to assist in their integration.

Secretory and soluble proteins that reside in the endomembrane system are typically synthesised with an N-terminal signal sequence, which directs them to the ER [1,2]. Once translocated to the ER lumen, the signal is then cleaved off (Figure 1A). Signal sequences are highly variable in sequence but typically are 12–30 residues in length and composed of an N-terminal region, often positively charged, a core of eight or more hydrophobic residues, and a short polar C-terminal domain which often contains helix-breaking glycine and proline residues, as well as amino acids with short-side chains at the -1 and -2 positions, the consensus site for the cleavage by the signal peptidase [2,3].

Integral membrane proteins can be classified based on their topology in the membrane. Single spanning membrane proteins (with just one trans-membrane (TM) domain) that have their N-termini in the ER lumen and C-terminus in the cytoplasm are termed type I, whereas those with their N-termini in the cytoplasm and C-terminus in the lumen are termed type II. In addition to this classification, a subclass of type I membrane proteins that possess very short luminal domains are called type III membrane proteins. Similarly, type II proteins with very short luminal domains are called tail-anchored proteins, reflecting the location of the TM domain close to the C-terminus (Figure 1A). Membrane proteins with multiple TM domains are termed polytopic membrane proteins.

Type I membrane proteins, similar to secretory proteins, are also targeted by cleavable signal sequences. In contrast, type II membrane proteins, including tail-anchored proteins, use their TM domain as a non-cleavable targeting sequence (Figure 1A) [4,5]. The same mechanism is also used by Type III membrane proteins. Targeting of polytopic membrane proteins is typically linked to the topology of the first TM domain; thus, where there is a large N-terminal luminal domain, targeting typically takes place via an N-terminal signal sequence. In contrast, where there is a short N-terminal luminal domain or where the N-terminus is in the cytosol, then, the first TM domain serves as a non-cleavable targeting signal as with type II membrane proteins. A key feature of all these targeting signals is their hydrophobic nature, and hence, a vital role of cellular targeting pathways is to prevent their aggregation while they are being targeted. 

## 2. ER Protein Translocases and Insertases

At the ER membrane, a number of protein translocases are able to either translocate proteins into the ER lumen or insert them into the lipid bilayer (Figure 1B). The canonical Sec61 translocase can perform both these functions with a large range of substrates. The more recently discovered GET insertase and EMC translocase can function independently of Sec61 but are limited to substrates with short luminal domains, while the recently discovered TMCO1 translocon functions in collaboration with Sec61 during polytopic membrane protein biogenesis [5,6,7]. Here, we shall focus on the targeting mechanisms which recognise substrates and deliver them to these translocases. The details of structure, function, and insertion mechanisms of these translocases are covered by a number of excellent recent reviews [5,6,8,9].

## 3. SRP-Dependent Targeting

**Figure 2:**
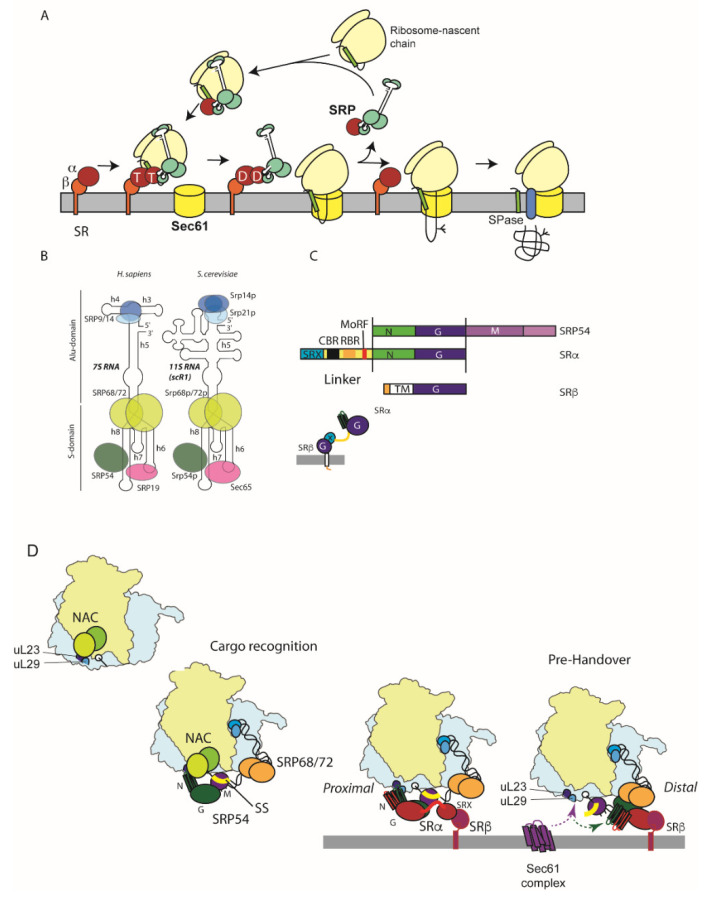
SRP-dependent targeting pathway: (**A**). Overview of the SRP targeting pathway: SRP can bind to ribosomes and scan for the presence of a signal sequence in the nascent chain as the N-terminus emerges from the ribosome exit tunnel. Engagement with the nascent chain with the SRP54 subunit (red) leads to a transient retardation in translation elongation and targeting to the ER membrane via an interaction with SRP receptor (SR). Complex formation between SRP and SR is driven by GTP (T) binding to SRP54 and SRα. The Sec61 translocase triggers release of signal sequence from SRP concomitant with hydrolysis of GTP to GDP (D). The ribosome binds Sec61 such that as translation resumes the nascent chain is threaded through the channel into the ER lumen where signal peptidase (SPase) can then remove the signal sequence. Finally, SRP and SR can dissociate, and nucleotide is released, permitting further rounds of targeting. (**B**). Domain organisation of yeast and mammalian SRP: Mammalian SRP is composed of a 7SL SRP RNA and six SRP proteins. SRP54, SRP19, SRP68, and SRP72 bind the RNA to form the S-domain whilst SRP9 and SRP14 form the Alu domain. Yeast SRP comprises a larger 11S RNA and homologues of SRP54, SRP19 (Sec65), SRP68, and SRP72 in the S domain and Srp14 and a dimer of Srp21 in the Alu domain. (**C**). Domain organisation of SRP54 and SRP receptor: SRP54 is comprised of a composite NG domain containing a four α-helical N- and GTPase (G) domain as well as a M-domain that comprises the RNA and signal sequence binding site. SRα has an N-terminal SRX domain that interacts with SRβ, a flexible linker with conserved CBR, RBR, and MoRF motifs and an NG domain closely related to that of SRP54. SRβ comprises an N-terminal TM domain and GTPase domain that interacts with SRX. (**D**). All ribosomes are associated with NAC, a nascent chain chaperone, which is bound at the exit site; SRP can transiently bind to the ribosome, positioning SRP54 at the exit site via an interaction with uL23 and uL29, allowing it to scan the emerging nascent chain for signal sequences. NAC aids the specificity of cargo loading of SRP54 with the signal sequence, which then stabilises ribosome binding. Initial interaction with SR at the ER membrane involves interaction of the SRP54 and SRα NG domains adjacent to uL23/uL29 (the proximal site) and is accelerated by the SRα linker MoRF motif binding to the SRP RNA when SRP54 is bound to a signal sequence. Subsequent dissociation and the NG domains from uL23/L29 and SR compaction move the NG domains to the distal site, where interactions involving SRP68, SR CBR motif, SRX, and SRβ stabilise its binding to form the ‘prehandover complex’. An interaction of the GTPase domains with SRP72 blocks GTP hydrolysis until Sec61 arrives. Movement to the distal site allows access to uL23/uL29 which is a key binding site for the Sec61 translocase and exposes the signal sequence and M-domain. This allows efficient transfer of the ribosome and nascent chain to Sec61 coordinated by concerted GTP hydrolysis by the NG domains.

The signal recognition particle (SRP) is a highly-conserved targeting machine present in all domains of life [10]. SRP is able to bind to translating ribosomes and can scan the emerging N-terminus of the nascent chain for the presence of signal sequences [11] (Figure 2A). Signal sequence recognition by SRP leads targeting of the ribosome together with the nascent chain to the ER membrane via the action of its cognate receptor (SRP receptor SR), an integral ER membrane protein which then facilitates the transfer of the ribosome and nascent chain to the Sec61 protein-conducting channel [12,13,14] (Figure 2A). SRP also induces a transient slowdown in translation, termed elongation arrest, which extends the time window where the ribosome-nascent chain complex remains competent to be targeted to Sec61 by the action of SR [15]. Once the nascent chain is released from the SRP–SR complex, the two can dissociate to allow further rounds of targeting.

The conserved core of the SRP targeting system, present in all domains of life, comprises the signal-sequence-binding protein of SRP, SRP54, associated with a conserved SRP RNA together with the SRα subunit of SR. SRP54 and SRα are both GTPases and possess closely related GTPase domains. In higher eukaryotes, SRP comprises a 300 nt 7S SRP RNA and six polypeptides organised in two domains: the S-domain which includes the conserved core as well as proteins SRP19, SRP68, and SRP72, and the Alu domain comprising proteins SRP9 and SRP14 [16] (Figure 2B). Yeast SRP possesses a slightly larger 11S RNA, a larger homologue of SRP19 (Sec65), and SRP9 is replaced with the related Srp21, and Srp14 is present as a homodimer [17] (Figure 2B). The S-domain of SRP is primarily involved with signal sequence recognition and accurate handover to the Sec61 translocation channel at the ER membrane facilitated by the interaction with SR. In contrast, the Alu domain is responsible for inducing the elongation arrest activity [18].

The SRP receptor comprises the conserved SRα subunit anchored at the ER membrane by single spanning-membrane protein SRβ, which also possesses a GTPase domain but which is more closely related to ARF and Sar1 than SRP54 and SRα [19] (Figure 2C).

Structural insight into SRP and its interaction with signal sequences and the ribosome have been provided by both X-ray crystallography and cryo-EM studies [11,20,21]. SRP54 is composed of two domains, the NG and M-domains, separated by a flexible linker which allows communication between the two domains [22] (Figure 2C). The N-domain comprises a four α-helical bundle which folds onto the GTPase domain [23]. The M-domain contains a helix-loop-helix structure, which allows SRP54 to bind to the SRP RNA and a hydrophobic groove which forms the binding site for signal sequences [24,25]. SRP19 does not contact SRP54 directly but also binds the SRP RNA and modifies its structure allowing SRP54 to bind [26].

Initial, low-affinity binding of SRP to ribosomes is independent of the signal sequence allowing it to dynamically scan ribosomes for the presence of signal sequences in the emerging nascent chain prior to high affinity binding upon recognition of the signal sequence [27,28]. Consistent with this observation, ribosome profiling experiments indicate that SRP can also be recruited to ribosomes prior to emergence of the signal sequence [29].

The S-domain of SRP binds to the ribosome on the 60S subunit at the exit site where the nascent chain emerges from the exit tunnel which conveys it from the peptidyl-transferase centre to the ribosome surface (Figure 2D) [20]. The N-domain of SRP54 contacts ribosomal proteins uL23 and L29 at one side of the exit tunnel, as well as three additional contact sites involving the SRP54 M-domain and SRP68/72 [11,20,30]. This positioning of SRP54 permits efficient scanning and capture of the signal sequence by the M-domain as it emerges [11,20,30,31]. Blocking access of SRP to uL23/L29 is known to lead to targeting defects in vivo [32].

The Alu domain of SRP contacts the interface of the large and small subunits at the translation elongation factor-binding site, thus rationalising the slowdown in translation elongation by antagonising factor binding [11,20] (Figure 2D). The C-terminus of SRP14 represents one of the contact sites, and its removal leads to targeting defects, which can be ameliorated by elevating SR concentration [20,33,34].

Slowdown of translation is not solely mediated by SRP; analysis of codon-optimality downstream from the targeting sequences revealed a prevalence of poorly translated codons distal to the signal sequence, which also contributes to slowed translation and replacement with more synonymous optimal codons, reducing targeting efficiency [35].

Careful kinetic and structural analysis of the SRP targeting cycle has provided key insight into the roles played by the two GTPases, SRP54 and SRα, in regulating targeting. SRP54 and SRα are both members of the SIMIBI family of GTPases which are characterised by a relatively low affinity for GTP compared to other small GTPases, such as Ras [36]. This is explained structurally by the presence of an additional insertion domain called the I-box which stabilises the nucleotide-free state [37].

SRα is organised into two domains, an N-terminal SRX domain, which folds together with the SRβ GTPase [38,39,40], and the SRP54-related NG domain, separated by a conserved flexible linker rich in positive charge (Figure 2C). SRP54 and SRα NG domains dimerise in a GTP-dependent manner, whereby the two bound nucleotides sit at the interface contacting one another in an anti-parallel organisation [41,42].

Once bound to a signal sequence, SRP54 can then engage the SRP receptor. This occurs in two steps, an initial low affinity binding where the SRP54 NG domain is still adjacent to ribosomal proteins, uL23 and uL29, termed the proximal site, followed by rearrangement to a high-affinity bound state where the NG-domains together move away from the ribosome surface together towards SRP68/72, termed the distal site, to form a ‘prehandover’ complex [43,44]. Initial complex formation at the proximal site is facilitated by GTP-dependent NG–NG domain interaction and is dramatically accelerated by the conserved molecular recognition feature (MoRF) in the unstructured SRα linker region, which contacts the SRP RNA [45]. This kinetic acceleration is dependent on cargo loading to SRP54, thereby strongly favouring complex formation upon recognition of the signal sequence [45]. Moreover, this is directly analogous to the role played by the SRP RNA in bacterial SRP–SR complex formation [46,47]. 

Following the initial complex formation by the two NG domains, there is destabilisation of the first α-helix in each of the two N domains and their associated loops which contact the ribosome [44,48]. This rationalises their dissociation from uL23/uL29 at the proximal site. Once the NG domains are dissociated, a compaction of SR brings the NG domain close to the SRX domain at the distal site [49]. This is stabilised by interactions between SRP68, elements of SRα NG, and X domains, as well as with SRβ [44]. The charged CBR motif in the SRα linker also makes an interaction with the distal site elements of the SRP RNA [44] (Figure 2D).

This prehandover complex is now primed to interact with the Sec61 translocon as the uL23/L29 site, which Sec61 also contacts, and is now accessible, as well as the signal sequence in the M-domain [30,43,44,50]. Whilst at the distal site, an interaction of the GTPase domain interface with the C-terminus of SRP72 delays hydrolysis of GTP by SRP54 and SRα until Sec61 is present [44,51].

The arrival of Sec61 leads to rearrangement of the prehandover complex which induces concerted GTP hydrolysis by SRP54 and SRα, thereby releasing the nascent chain from SRP and allowing its transfer together with the ribosome to the Sec61 complex [14,43,52]. Following GTP hydrolysis, SRP and SR can dissociate to undertake further rounds of targeting [53]. 

Recent studies have shown that SRP alone is unable to discriminate with high precision cognate and near-cognate (non-functional) signal sequences. Another ribosome-associated biogenesis factor, NAC, also needs to collaborate with SRP to enhance fidelity [54] (Figure 2D). NAC binds to all ribosomes and contacts overlapping regions at the exit site to where SRP binds [20,55,56,57]. It is also able to initially insert a domain deep into the exit tunnel which would prohibit interactions of factors with non-translating ribosomes; subsequent displacement of this domain by the nascent chain has been proposed to lead to dynamic rearrangement of NAC which appears important for the correct triage of nascent chains to SRP in the case of secretory and membrane proteins and RAC and Hsp70 in the case of cytosolic proteins [57,58]. Hence, the increased fidelity in signal sequence recognition in the presence of NAC is entirely consistent with this model.

Proteomic approaches in yeast have used ribosome profiling to identify substrates and binding sites for SRP on translating polysomes [29]. SRP is associated mainly with ribosomes translating integral membrane proteins and to a lesser extent proteins with N-terminal signal sequences [29]. This parallels similar studies in bacteria which also show SRP is mainly involved in membrane protein biogenesis [59]. These findings are also supported by SRP-substrate dependency as measured proteome-wide by loss of ER mRNA association upon rapid depletion of SRP [60]. This analysis revealed a similar SRP-dependency profile that is again biased towards integral membrane proteins [60]. The ribosome profiling experiments also revealed that whilst for some substrates SRP recruitment occurs as the targeting sequence emerges from the ribosome as expected in the canonical model of SRP function, in many cases SRP is recruited earlier, often well before the signal sequence/anchor has been synthesised [29]. This non-canonical binding of SRP is still dependent upon the ribosome and in some cases requires features of the 3′ UTR, but it is not presently known how preferential pre-delivery of SRP to these substrates occurs and if it requires specific mRNA binding proteins [29]. Interestingly, it was recently shown that the ribosome-associated factor, Hel2, which binds to collided ribosomes that arise where stalling occurs, was shown to bind to membrane protein polysomes in a pattern that strongly overlaps with that of SRP [61].

In the absence of SRP, substrates that are usually delivered to the ER can become aggregated [62] or are instead mistargeted to mitochondria causing disruption to mitochondrial function [56,60], a phenotype also observed if Hel2 is disrupted [61]. Hence, another key function of SRP is to prevent promiscuous misbehaviour of hydrophobic targeting signals.

## 4. SND-Targeting Pathway

SRP is not the only co-translational ER-targeting pathway. Studies in yeast investigating targeting of GPI-anchored proteins identified the SND pathway, comprising the cytosolic factor SND1 associated with the ribosome and ER-membrane associated SND2/3 proteins [63]. Exploitation of this pathway is not exclusive to GPI-anchored proteins but rather membrane proteins with centrally located TM domains. The SND pathway also operates in mammalian cells, although to date, only the homologue of SND2 has been identified [64]. As seen in yeast, human GPI-anchored proteins also use SND2 for their targeting to the ER [65]. Detailed molecular details of the SND-targeting pathway await further investigation.

## 5. Sec62-Dependent Targeting

Work in yeast showed that ER targeting of many secretory precursors is independent of SRP and SR but rather requires the ER membrane protein, Sec62 [66], which forms a larger SEC complex along with the core Sec61 translocase as well as Sec63, Sec71, and Sec72 [67]. Sec62 is dispensable for targeting of substrates that use SRP and SR [68,69]. Also, in contrast to the SRP pathway, targeting can be uncoupled from translation [70]. Indeed, the SEC complex cannot bind ribosomes directly [71,72], rationalised by recent structures of the SEC complex which show the ribosome-binding sites on the core Sec61 components are all occupied by the additional components of the SEC complex [73].

Cytosolic Hsp70s and Hsp40 are required to maintain substrates in a translocation-competent state [74,75], and Sec71/72 have been shown to be receptors for Hsp70 family chaperones [76]. Properties of the signal sequence largely determine which targeting pathway a substrate will use, with more hydrophobic sequences exclusively using the SRP-pathway and less hydrophobic ones using Sec62, while intermediate hydrophobic ones can access both [69].

Although Sec62-dependent translocation can be uncoupled from translation, proteome-wide proximity labelling experiments in yeast have shown that most substrates are translocated co-translationally, likely driven by engagement of a pioneer ribosome-associated signal-sequence-bearing protein with the SEC complex before its translation has been completed [77]. This will bring the polysome close to the membrane such that subsequent nascent chains are also highly likely to engage the SEC translocon before their synthesis is complete [77].

Homologues of Sec62 as well as Sec63 (but not Sec71/72) also exist in higher eukaryotes [72,78]. Unlike its yeast counterpart, mammalian Sec62 has an additional ribosome-binding domain [79]. They have mainly been implicated in the targeting and translocation of short-secretory proteins (<100 amino acids), such as the insect proteins, preprocecropin A and prepromelittin [80,81,82,83], and mammalian proteins, such as insulin, apelin, and statherin [81,84]. These proteins are so short that they are released from the ribosome before SRP can effectively engage with them and so are targeted post-translationally. In the case of preprocecropin A, an interaction with calmodulin is important to maintain the protein in an insertion-competent state [85]. This is a calcium-dependent phenomenon that can occur already at resting cytosolic calcium levels [85]. Proteomic approaches in human cells have identified 199 proteins whose biogenesis is negatively affected by loss of Sec62 [86]. Not all of these are short secretory proteins, but as in yeast, they typically possess signal peptide or trans-membrane domains with lower hydrophobicity [86]. This may reflect a role of Sec62 in their SRP-independent targeting and/or a requirement of Sec62 at the translocon at the later stages of translocation following targeting via SRP and SR [87,88]. Overall, targeting sequences in mammalian cells have higher hydrophobicity than in yeast, which likely reflects the difference in bias in SRP versus Sec62-dependence in the two systems [85].

## 6. GET-Targeting Pathway

**Figure 3:**
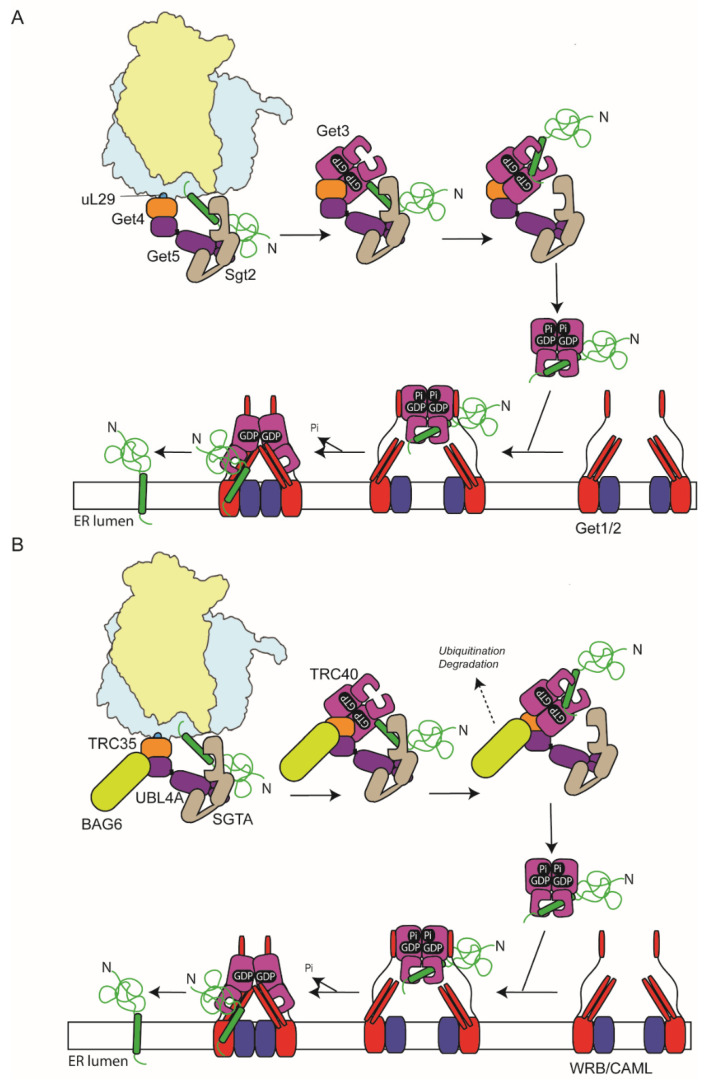
GET-targeting pathways for tail-anchored proteins in yeast and mammals. (**A**) In yeast, the Get4/5 complex is able to bind to the ribosome via an interaction of Get4, close to the exit site and uL29. Get5 can then recruit Sgt2, which is able to capture a tail-anchor TM domain as it emerges from the exit tunnel. The Sgt2-Get4/5-TA protein can be released from the ribosome and then the TA protein can be directly transferred to Get3, a homodimeric ATPase, which is recruited in the ATP bound state. Release from Get4/5 leads to ATP hydrolysis to ADP and P_i,_ which remain bound, altering the conformation of the TA-binding domain such that the TA remains bound but can now bind the membrane receptor Get1/2. Get1/2 is from a dimeric complex embedded in the ER membrane that can assemble to a tetramer. Cytosolic elements of Get1 bind Get3 and allow insertion of a coiled coil, like a wedge into the ATPase domain interface of Get3, which triggers phosphate release and reorganisation of the TA-binding regions such that the TA is now released into a hydrophilic cavity between Get3 and the inter-membrane regions. This directs the TA to the insertase formed by the membrane-spanning regions of Get1/2. (**B**) In mammals, initial TA capture involves SGTA and the BAG complex (formed by Bag6, Ubl4A and TRC35). Again, this occurs co-translationally at the ribosome. TA proteins are then efficiently transferred from SGTA to TRC40; BAG6 can additionally triage hydrophobic, non-TA proteins to the ubiquitin proteasome system for degradation. As with the yeast TRC40 homologue, Get3, the TRC40 ATPase cycle controls recruitment and transfer of the TA protein from SGTA and subsequent delivery to the membrane insertase formed by the Get1/2 homologues, WRB/CAML.

Proteins which possess a C-terminal TM anchor (tail anchor [TA]) are unable to access the classical co-translational SRP-targeting machinery as they are released from the ribosomes before the TA sequence has emerged from the exit tunnel [89]. Rather they use the distinct GET (guided-entry of tail-anchor proteins) machinery for their delivery to the ER [4,90,91]. In yeast, they are first bound by the cytosolic targeting factor, Sgt2, which acts as a pre-loading complex and are then transferred to Get3, facilitated by its accessory proteins, Get4 and Get5 [92,93] (Figure 3A). The Get3-targeting factor can then deliver the proteins to the Get1/Get2 ER membrane heterodimer, which acts as both a receptor for Get3 and also a membrane insertase [92,94]. In higher eukaryotes, homologous of all these key components are present: SGTA (Sgt2), TRC40 (Get3), TRC35 (Get4), Ubl4A (Get5), and CAML/WRB (Get1/Get2) [95,96,97,98] (Figure 3B). In addition, an extra factor, BAG6, is present in a complex with TRC35 and Ubl4A (called the BAG6 complex) and acts to triage hydrophobic clients between TRC40 and the ubiquitin-proteasome system [99,100]. While *bona fide* ER TA proteins are delivered faithfully to TRC40, mis-localised membrane and secretory proteins are also bound by the BAG6 complex and directed for ubiquitination and disposal via the proteasome [99]. Hence, BAG6 has a role in both targeting fidelity and proteostasis.

Initial capture of TA sequences by the SGTA pre-loading complex occurs at the ribosome [97] (Figure 3B). Furthermore, a recent in vitro study indicated that SGTA can be pre-recruited to the translating ribosome, prior to the emergence of a hydrophobic sequence from the exit tunnel, thereby allowing co-translational capture of TA segments for subsequent handover to TRC40 [101]. BAG6, TRC40, and TRC35 are also ribosome associated, indicating this step also occurs at the ribosome [97]. In the case of the Sec61β, delayed termination of translation due to pausing at the stop codon also likely enhances TA capture by TRC40 [97,102].

Interestingly, SRP-dependent targeting sequences could be similarly engaged by SGTA, this may reflect a holdase function prior to SRP binding, or a mechanism for targeting signal sequences that SRP fails to recognise for degradation via BAG6 [101].

Studies in yeast have shown that Get4 and Get5 bind to non-programmed ribosomes with high affinity mediated by an interaction of Get4 with the ribosome close to ribosomal proteins uL29 and uL26 at the exit site and that this enhances recruitment of Sgt2 [103]. Consistent with the observations with SGTA, the presence of a TM inside the ribosome exit tunnel enhances ribosome binding of Get4/5 and Sgt2 as has also been seen previously with SRP [103,104,105]. The ribosome-binding site of SRP and Get5 overlap [20,30,103], and once SRP binds to a SA/SS that has emerged from the exit tunnel, Get4/5 binding is inhibited [103]. The Hsp70 ATPase Ssa1 has also been shown to be important for efficient transfer of TA proteins to Sgt2 in the absence of the ribosome; thus, it may rescue any TA clients that fail to be captured by Sgt2 at the ribosome [106].

Get3/TRC40, the central player of the GET pathway is a homodimeric ATPase, composed of a P-type ATPase domain and alpha-helical domain, which is involved in TA binding [107,108]. The ATPase cycle of Get3 controls the conformation of the TA-binding region, such that in the ATP-bound state the homodimer forms a fully closed state that forms a composite hydrophobic groove that can accommodate a 20 amino acid long hydrophobic helix [107,108]. Hydrolysis of ATP to the ADP bound state partially disrupts the groove but still allows the TA to remain bound, while in the nucleotide-free state the composite groove is completely disrupted [90].

Get3 is recruited to Get4 in the ATP-bound state, which inhibits ATP hydrolysis and promotes direct transfer of the substrate from Sgt2 to the hydrophobic groove in Get3 [109,110]. A dynamic α-helix in Get3 that forms a lid facilitates transfer and prevents delivery of hydrophobic helices that were not pre-bound to Sgt2 [111]. Binding of the substrate to Get3 stimulates ATP hydrolysis releasing it from the preloading complex and allowing it to interact with the Get1/2 insertase at the ER membrane in the ADP-bound conformation [110,112,113].

Get1 and Get2 both possess cytoplasmic domains that extend from the membrane and engage Get3. Upon binding, a coiled-coil domain within Get1 inserts between the subunits of Get3 and thereby stabilises the nucleotide-free conformation, triggering substrate release [113,114,115].

Recent structures of the complete Get1/2/3 complex, including the TM regions of Get1/2, show that Get1/2 forms a hetero-tetramer with symmetric recruitment of Get3, where both its subunits contact separate Get2 subunits [116]. Furthermore, mutations at the hetero-tetramer interface decrease the efficiency of membrane insertion, suggesting the tetramer is the active form. However, it may still be possible for insertion to occur via a Get1/2 heterodimer as has also been proposed [112]. A hydrophilic cavity is formed between the trans-membrane region and Get3. Furthermore, a hydrophobic helix (a3′) in Get1 forms a gate adjacent to the TA-binding region of Get3 and the cavity such that as the cargo is released, the a3′ helix is displaced forming a wall to direct the cargo into the cavity [116].

Despite the essential function of many tail-anchor proteins, loss of the GET machinery is not lethal to yeast cells, indicating functional redundancy [94]. In particular, the alternate co-translational SND pathway can also accommodate TA proteins in the absence of GET components [63]. This is also reflected in mammalian cells; TA proteins with moderately hydrophobic TMs can be recognised by calmodulin and then targeted to the EMC complex [85,117,118]. Hsp70s and ubiquilin family proteins can similarly maintain TA proteins in a soluble state in the cytosol competent for insertion [4], whilst the more hydrophobic TA protein, Sec61β, can engage both SRP and TRC40 as well as the mammalian SND complex [64,97,119].

## 7. EMC Translocase

The alternative EMC translocase has been implicated in the biogenesis of both tail-anchor membrane proteins, as mentioned above, and those with a type III orientation. In both cases, the substrates possess a targeting membrane domain that is positioned close to the C- and N-termini, respectively, such that only a small hydrophilic domain has to traverse the bilayer [117,118,120,121]. It can also collaborate with Sec61 during the biogenesis of some polytopic membrane proteins, particularly those with a short N-terminal luminal domain, akin to type III proteins, such as many GPCRs [122,123]. Targeting of type III membrane proteins in both yeast and mammalian cells involves SRP and SR [60,69,121,124], yet the manner in which they are handed over from the ribosome–SRP–SR complex to EMC remains unknown. Intriguingly, insertion of type III proteins in mammalian cells is insensitive to Sec61-inihbitors, including Ipomoeassin-F, which blocks translocation of all other known substrates via the Sec61 channel [121]. In contrast, depletion of Sec61 does impact type III insertion [121], suggesting Sec61 might play a non-canonical role, perhaps facilitating the release from the SRP–SR complex and handover to EMC. 

## 8. Defective ER Targeting and Human Disease

A number of patient mutations have been identified which map to components of the ER protein-targeting machinery. Point mutations in SRP54 are associated with severe neutropenia and Shwachmann–Diamond syndrome-like symptoms, which affect development and function of tissues with high secretory activity, such as the pancreas, as well as skeletal and neurodevelopmental defects [125,126]. While some of the mutations are attributable to loss of function and haploinsufficiency, others, including T115A, T117∆, and G226E, have a dominant phenotype. All mutations to date map to the G-domain where they are implicated to impact either nucleotide binding or overall structure. Several of the mutants have been analysed in detail (T115A, T117∆, and G226E) revealing structural changes to the core GTPase, which impair GTP binding and prevent complex formation of isolated SRP54 and SRα NG domains [127]. A more detailed analysis of the G226E mutant when assembled in SRP in the context of SR and the RNC reveals that while initial SRP–SR complex assembly can occur, it becomes locked in an RNC–SRP–SR intermediate that cannot relocate the NG domains from the proximal to distal position, thereby rationalising the dominant negative phenotype associated with the mutation [49].

Using a zebrafish model, the severe neutropenia-associated phenotypes associated with autosomal dominant mutations (T115A, T117∆, and G226E) are phenocopied along with pancreatic dysfunction [128]. Furthermore, the neutropenia phenotype has been linked to impaired splicing of the *XBP1* transcription factor required for the unfolded protein response and which is spliced in an unconventional manner by *IRE1* following its membrane targeting by SRP [129]. Loss of Xbp1 in the zebrafish also shows a similar neutropenia phenotype, consistent with this being a key driver of the disease phenotype [128].

Disease mutations are not limited to SRP54; a recently identified biallelic mutant of SRP68 that leads to loss of exon1 is also associated with neutropenia and Shwachmann–Diamond-like symptoms [130], whilst SRP72 mutants have been linked to aplastic anemia (AA) and myelodysplasia (MDS) [131].

As well as mutations in SRP, disease mutations that impact protein targeting are also linked to mutations in targeting sequences in client proteins [132]. First shown with synthetic mutations in the well-studied model signal sequence from bovine preprolactin, a reduction in the length of the hydrophobic core led to loss of SRP binding and instead the nascent chains interacted with Argonaut2 (Ago2). As well as blocking translocation in vitro, this also promoted rapid degradation of the mRNA in vivo in a quality control pathway termed RAPP (Regulation of Aberrant Protein Production) [133]. A number of disease-linked mutations in signal sequence mutations also trigger this pathway in response [132,134], for example in granulin linked to fronto-temporal lobal degeneration [134,135], aspartylglucosaminidase in aspartylglucosaminuria [134,136], UDP-glucuronosyltransferase in Crigler–Najjar disease [134,137], and cathepsin K in pycnodysostosis [134,138].

### Future Outlook

Despite more than 40 years of research into ER protein-targeting, questions clearly remained unanswered. In particular, the SND complex remains only very basically characterised in terms of mechanism and structural characterisation, and the identity of the mammalian components beyond hSnd2 remain elusive. While the canonical mode of action of SRP recruitment has been studied in much detail, the observed recruitment of SRP to polysomes translating membrane proteins prior to synthesis of the signal sequence/anchor remains poorly understood, likewise the recent link between SRP binding and the Hel2 protein. Mechanistic understanding of the role NAC plays at the ribosome has been hampered by lack of structural images until very recently, and there is much scope to understand how NAC functions to enhance SRP targeting. Proteomic profiling of substrate and targeting factor interaction in yeast has proved highly informative, and extending this to the more complex mammalian system should shed new light on the interplay between targeting pathways. Hence, there is still much to find out for the future. 

## Data Availability

Not applicable.
